# Modeling strength characteristics of basalt fiber reinforced concrete using multiple explainable machine learning with a graphical user interface

**DOI:** 10.1038/s41598-023-40513-x

**Published:** 2023-08-12

**Authors:** W. K. V. J. B. Kulasooriya, R. S. S. Ranasinghe, Udara Sachinthana Perera, P. Thisovithan, I. U. Ekanayake, D. P. P. Meddage

**Affiliations:** 1 Department of Civil Engineering, Sri Lanka Institute of Information Technology, Malabe, Sri Lanka; 2 Department of Computer Engineering, University of Peradeniya, Kandy, Sri Lanka; 3 Department of Civil Engineering, University of Moratuwa, Moratuwa, Sri Lanka

**Keywords:** Civil engineering, Mechanical properties, Engineering

## Abstract

This study investigated the importance of applying explainable artificial intelligence (XAI) on different machine learning (ML) models developed to predict the strength characteristics of basalt-fiber reinforced concrete (BFRC). Even though ML is widely adopted in strength prediction in concrete, the black-box nature of predictions hinders the interpretation of results. Among several attempts to overcome this limitation by using explainable AI, researchers have employed only a single explanation method. In this study, we used three tree-based ML models (Decision tree, Gradient Boosting tree, and Light Gradient Boosting Machine) to predict the mechanical strength characteristics (compressive strength, flexural strength, and tensile strength) of basal fiber reinforced concrete (BFRC). For the first time, we employed two explanation methods (Shapley additive explanations (SHAP) and local interpretable model-agnostic explanations (LIME)) to provide explanations for all models. These explainable methods reveal the underlying decision-making criteria of complex machine learning models, improving the end user's trust. The comparison highlights that tree-based models obtained good accuracy in predicting strength characteristics yet, their explanations were different either by the magnitude of feature importance or the order of importance. This disagreement pushes towards complicated decision-making based on ML predictions which further stresses (1) extending XAI-based research in concrete strength predictions, and (2) involving domain experts to evaluate XAI results. The study concludes with the development of a “user-friendly computer application” which enables quick strength prediction of basalt fiber reinforced concrete (BFRC).

## Introduction

Basalt fibers are obtained from basalt rocks through the process of melting. It is possible to produce fibers from Basalt rocks by finely dividing them. Basalt fiber is an inorganic, bio-degradable, non-metallic material. It is widely used to improve the tensile capacity of concrete because of its strong tensile property. The production process of basalt fiber is cost-effective since it does not need any additive mixing. Basalt fibers show exceptional tensile strength over E-glass fiber, higher breaking strength over carbon fiber, along with better resistance to chemical attacks, fire, and impact loads^1^. These properties have led the research community to focus on applying basal fiber as an innovative structural reinforcement material thus can produce reinforced concrete.

Compressive strength, tensile strength, and flexural strength of concrete are considered basic strength characteristics of concrete^2,3^. To fully elucidate the impact of basalt fibers, several studies have been carried out to determine the mechanical properties of BFRC^4^. Meyyappan and Carmichael^5^ used different volume fractions of basal fibers and observed that both split tensile strength and compressive strength increase in the presence of basalt fiber. However, the variation reached an optimum at 1% volume fraction and subsequently depicted a declining trend. The increase in compressive strength at the optimum fraction was 11.5% and 18.2% for split tensile strength compared to the control sample. Chen et al.^6^ used the content of basalt fiber as a variable to study the effect on the mechanical properties of BFRC. Jalasutran et al.^7^ made similar arguments by investigating the mechanical properties of BFRC. They observed the strength characteristics improve as a result of basalt fiber content. However, the addition of basalt fiber has caused non-linear variation in strength characteristics^5,8,9^. As a result, the prediction of strength characteristics of BFRC is relatively complicated compared to conventional concrete and requires an iterative experiment process to study the relationships progressively. Accurate estimation of strength characteristics is highly imperative to structural design and optimizations. On the other hand, existing research studies have derived results based on laboratory experiments which are highly time-consuming, laborious, and costly. As an alternative approach, analytical methods such as machine learning (ML) techniques can be used to predict the strength characteristics of BFRC.

Recent trends on applying ML into concrete strength prediction has enabled researchers to explore performances of various concrete mixes. According to Golafshani et al.^10^, ML techniques can efficiently predicts compressive strength of eco-friendly concrete which can further optimized using particle swarm optimization. A latest study of Ghanbari et al.^11^ found that artificial neural network (ANN) is well-performing over other ML techniques that can predict the compressive strength of waste-based concrete mixes. Basaran et al.^12^ used the Gaussian process regression (GPR) model to predict the FRP (Fiber reinforced plastic)-concrete bond with an accuracy of 95% and with a 0.14 standard deviation. Iqbal et al.^13^ proposed an empirical-based new strategy to predict tensile strength using artificial neural networks (ANN), adaptive neuro-fuzzy inference systems (ANFIS), and gene expression programming (GEP) with good accuracy (R > 0.8) in all three models. The study conducted by Salami et al.^14^ explored the nonlinear properties of compressive strength in ternary composite concrete. They employed coupled simulated annealing (CSA) as an optimization algorithm in combination with the least squares support vector machine (LSSVM) to forecast compressive strength with an impressive R^2^ value of 0.954. Zhang and Aslani^15^ proposed an artificial neural network (ANN) model to predict the compressive strength of lightweight aggregate concrete based on UPV (Ultrasonic Pulse Velocity) under different conditions which resulted in a maximum ${\mathrm{R}}^{2}$ of 0.988, and a minimum of 0.736. By leveraging complex potential physical phenomena like mechanical properties, concrete composition, and experimental processes, Liu et al.^16^ constructed a model utilizing an ANN for predicting the chloride ion diffusion coefficient in concrete. Güçlüer et al.^17^ used ML models (ANN, Decision tree (DT), Support vector regression (SVR), and Linear regression) to predict 28-day compressive strength. DT model was selected as the best model with an R^2^ of 0.86. Kang et al.^3^ developed 12 machine-learning models to predict the compressive and flexural strength of steel fiber-reinforced concrete. Their gradient boosting (GB) model (MAE = 1.18) and extreme gradient boosting (XGB) model (MAE = 1.25) obtained superior performance compared to the remaining models. Nguyen et al.^18^ employed ANN, SVR, GB, and XGB to predict the compressive strength of concrete. They argued that GB regression and the XGB model performed better compared to ANN and SVR models. Feng et al.^19^ used an adaptive boosting (ADABoost) model to predict the compressive strength of concrete and the model achieved an R^2^ of 0.982 with MAE = 1.64. Similar studies were conducted by Asteris et al.^20^ and DeRousseau et al.^21^ to predict the compressive strength of concrete. Fang et al.^22^ used an image segmentation method to investigate the effect of pore structure on the split tensile strength of cellular concrete. Malami et al.^23^ used a neuro-fuzzy hybrid model composed of, an extreme learning machine (ELM), an adaptive neuro-fuzzy inference system (ANFIS), a multi-linear regression model (MLR), and SVR to study the impact of carbonization on reinforced concrete durability (R $\ge$ 0.96). Ashrafian et al.^24^ have developed an evolutionary-based ML model which give promising prediction of post-fire mechanical properties of green concrete. Recently, Li et al.^8^ used machine learning methods to predict the compressive strength of BFRC. They proposed random forests to predict the compressive strength and later used the Kernel extreme learning machine with genetic algorithms (KELM-GA) to perform the same task^9^. They argued that KELM-GA outperformed the models such as ANN, SVR, and Gaussian process regression (GPR). Behnood et al.^25^ used ML to model the elastic modulus, the flexural, compressive, and split tensile strength of concrete. Ashrafian et al.^26^ have shown that ML can accurately predict apparent surface chloride concentration of structural concrete in a marine environment. On these ML approaches to predict the mechanical properties of concrete, Chaabene et al.^27^ conducted a comprehensive review. They reported that conventional machine learning (ML) models do not explain the model despite the higher accuracy of results prediction. The model interpretation is important for structural engineering applications due to three reasons; (1) to identify interactions between inputs and underlying reasoning, (2) to establish the end-user’s and domain experts’ trust on ML, (3) to explain proposed methods to the non-technical community specially with less understanding about machine learning. Hence, the boundary of ML research has pushed towards revealing characteristic of black-box predictions.

Explainable Artificial Intelligence (XAI) seeks to address the previous limitation of insufficient model interpretation^28^. XAI provides the reasoning behind how a particular prediction is made^29^. Hence, XAI is very popular in the context that dealing with high stakes^30^. XAI converts models from black box to glass box (transparent) models by exposing underlying reasoning^31–38^. Recently, several researchers started using XAI to interpret the concrete strength characteristics obtained from machine learning models. Mostly, they used only Shapley additive explanations (SHAP) to interpret the models). Table 1 summarizes the application of XAI in concrete strength prediction research in recent years.

**Table 1:** Related work that used XAI methods to explain the strength characteristics of concrete.

References	Prediction	Models used	Explanation method	Explanations provided
^36^	Chloride migration	DT RF ET ADBoost GB XGB	SHAP for XGB	Feature importance/sensitivity study
^32^	Compressive strength of high-strength concrete	XGB	SHAP for XGB	Global explanation
^35^	Compressive and tensile strength of concrete	LASSO RF ANN XGB SVR	SHAP for XGB	Global explanations Feature interaction plot
^34^	Compressive strength of concrete	EBM RF DT XGB	EBM (model itself)	Global explanation Feature dependency Local explanation
^38^	Compressive strength of concrete	ANN SVR ADBoost CNN	Sensitivity analysis on CNN	Feature dependency
^37^	Compressive strength of sustainable concrete	SVR	SHAP on SVR	Global explanation Feature dependency
^2^	Compressive strength of high-strength concrete	XGB ADBoost ET DT LGB LKRR	SHAP on XGB	Global explanation Local explanation
Present study	Compressive, flexural, and tensile strength of basalt fiber reinforced concrete	DT GB LGB	SHAP and LIME on all three models	Global explanation Local explanation Feature dependency

Moreover, the authors have also discovered that XAI explanations for concrete strength predictions demonstrate a satisfactory alignment with the empirically established correlation between concrete parameters and strength.

With such advantages, XAI can be readily used to model the relationships of concrete with various constituents. However, these XAI-based studies highlight a common research gap. They only used a single explanation method on their best-performed model. Under such circumstances, they solely relied on the explanation provided on the best-performed model. However, the authors believe that explanations would change (substantially or moderately) based on the learning algorithm. Besides, two XAI methods would not provide the same explanations even for the same ML model. In other terms, the difference in explanations would create more complicated decision-making.

Moreover, no study is found which has employed XAI to explain strength characteristic (compressive, tensile, and flexural) prediction models of BFRC. By considering this significant research gap, the authors aimed to investigate the relationship between strength characteristics and the constituents of BFRC obtained from supervised tree-based ML then to perform a comparative analysis on all ML models using XAI. The authors used both Shapley additive explanation (SHAP) and local interpretable model agnostic explanations (LIME) for the present study. This novelty is summarized in Table 1 of this paper. Finally, a ready-to-use computer application was developed with a graphical user interface to predict strength of BFRC. This application can be utilized for quick and reliable strength prediction for various industry application. The significance of this study can be summarized into five contexts: (a) use of machine learning to predict compressive, tensile, and flexural strength of BFRC (b) use of XML to elucidate the strength characteristics of BFRC, (c) use of both LIME and SHAP algorithms as explainable methods, (d) For the first time, we used both SHAP and LIME on all ML algorithms and (e) Developed a user-friendly GUI to predict the mechanical strength of BFRC.

## Methods and data

### Explainable artificial intelligence (XAI)

XAI can be primarily categorized into two parts based on the model complexity^39^. Model explanations are convenient for simple models such as linear regression, and decision trees. These models can be explained directly how input features affect the output. Complex model structures usually improve the accuracy of ML modeling compared to a simple model. However, a model with complex structures cannot be easily explained^40^. Post-hoc explanation methods are necessary in such cases to provide human-understandable explanations for the predictions made by complex ML models^41^. For the present study, we used SHAP^42^ and LIME^43^.

#### Shapley additive explanations (SHAP)

Lundberg and Lee^42^ proposed SHAP as a solution for the interpretability of complex models. SHAP reframes the Shapley value problems on how members of a coalition contribute to a coalition value. SHAP considers the contribution of every individual feature which influences the target output^44^.

#### *Local interpretable model-agnostic explanations* (*LIME)*

LIME uses local linear models of ML outcomes to explain parts of complex machine-learned response functions^45^. LIME is used around an area of interest. Therefore, it explains part of an ML model's behavior and a local Interpretability Model rather than a global one^46^. LIME is powerful when it focuses on regions of the model that exhibit linear behavior, yet it can fail in areas of non-linearity.

### Machine learning (ML) models

For this study, we used tree-based models, namely, decision trees, gradient boosting trees and light gradient boosting trees. The first model is the basic form of tree models while the latter two models are ensemble (combination of multiple decision trees) models. According to Güçlüer et al.^17^ decision trees are less complex and accurate compared to complex models such as artificial neural networks (ANNs). Generally, optimization of tree models is also convenient compared to neural networks. Moreover, ensemble models based on decision trees have showcased good performance in predicting the compressive strength of fiber reinforced concrete^3^ and high strength concrete^2^ compared to conventional models.

#### Decision tree (DT)

DT divides a complex problem into several simple forms through the learning process. This results in a convenient interpretation of the model^47^. DTs are highly transparent and are formed based on reasonable assumptions^48^. Multiple regression and recursive partitioning are the basis for DT formation. The recursive splits continue until the final criterion is met^49^. To enhance the generalization of the model and reduce its complexity, a reduction sequence is carried out, whereby each leaf node represents a straightforward regression model.

#### Gradient boosting regression tree (GB)

Breiman et al.^50^ introduced Classification and regression trees (CARTs) where Ding et al.^51^ showed the usage of CARTs for problems in classification and regression. The GB algorithm is the combination GB algorithm introduced by He et al.^52^ and CARTs. CART has proven records for better performance over many ML models which can capture nonlinear correlations without the need to specify the distribution features of the model^52^. Gradient boosting is a technique that seeks to convert weak learners into strong learners. The algorithm works by progressively integrating weak learners into the model until a strong learner is achieved through the integration of multiple weak learners.

#### Light gradient boosting machine (LGB)

LGB utilizes tree-based learning algorithms on the GB framework. Gradient boosting is an ensemble technique where you have multiple models to make predictions, where the obtained errors from a model are, used to train the next model and at the end, all the models are combined to take the final model. Some of the advantages of using LGB are better accuracy, fast training speed, high efficiency, the capability of handling large-scale data, and lower memory usage^53^.

### Data description

Data for strength characteristics of BFRC concrete was obtained from the Mendeley repository^54^. The data set consists of three sets each for: (a) compressive strength, (b) flexural strength, and (c) split-tensile strength. Each set consists of 10 independent variables and a single dependent variable. All strength characteristics represent 28-day strength. Annex A1 represents descriptive statistics of all three data sets and Annex A2 depicts the pairwise correlation coefficient between independent and dependent parameters.

### Performance evaluation

For the model training and validation, we used 70% training and a 30% testing split. This split was chosen based on a trial-and-error approach by considering the combination of possible splits that gives the highest accuracy. Each ML model is assessed for its performance in both training and testing processes. Model training and hyperparameter optimization was performed simultaneously to improve the model performance. For the model performance evaluation, we suggested the following indices (Eqs. 1–4).

1 $${\mathrm{R}}^{2}=\frac{{\sum }_{\mathrm{i}=1}^{\mathrm{N}}{({\mathrm{P}}_{\mathrm{i}}-{\mathrm{O}}_{\mathrm{i}})}^{2}}{{\sum }_{\mathrm{i}=1}^{\mathrm{N}}{({\mathrm{P}}_{\mathrm{i}}-{\overline{\mathrm{O}} }_{\mathrm{i}})}^{2}}$$

2 $$\mathrm{MAE}=\frac{{\sum }_{\mathrm{i}=1}^{\mathrm{N}}\left|{\mathrm{O}}_{\mathrm{i}}-{\mathrm{P}}_{\mathrm{i}}\right|}{\mathrm{N}}$$

3 $$\mathrm{MSE}=\frac{{\sum }_{\mathrm{i}=1}^{\mathrm{N}}{{(\mathrm{O}}_{\mathrm{i}}-{\mathrm{P}}_{\mathrm{i}})}^{2}}{\mathrm{N}}$$

4 $$\mathrm{Fractional\,Bias}= \frac{2(\overline{{\mathrm{P} }_{\mathrm{i}}}-\overline{{\mathrm{O} }_{\mathrm{i}}})}{(\overline{{\mathrm{P} }_{\mathrm{i}}}+\overline{{\mathrm{O} }_{\mathrm{i}}})}$$

where ${\mathrm{P}}_{\mathrm{i}}$—Prediction value, ${\mathrm{O}}_{\mathrm{i}}$—Experimental value, ${\overline{\mathrm{O}} }_{\mathrm{i}}$—Mean value of the experimental set, $\overline{{\mathrm{P} }_{\mathrm{i}}}$—Mean value of the predicted set.

### Methodology of the study

**Figure 1:**
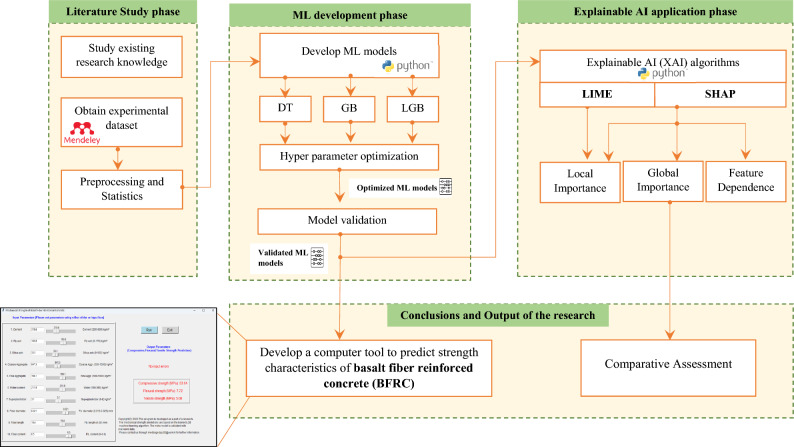
Workflow of the study.

The methodology of the research is illustrated in Fig. 1 which consists of three main phases. For ML models, the authors used SciKit Library^55^. The application of XAI (for all models) and comparative analysis outline the novelty of this study.

## Results and discussion

In order to optimize the hyperparameter, we used a grid search algorithm. Grid search creates combinations of hyperparameters and creates a model for each combination and evaluates their performance. It will provide the optimum set of values for each hyperparameter for a respective model. Annex A3 denotes the optimized hyperparameters used for each model. All the performance indices were separately calculated for model-wise and training and testing. Those performance indices are summarized in Table 2.

**Table 2:** Performance indices of tree-based regression models.

Mechanical strength	Performance index	DT training	DT testing	GB training	GB testing	LGB training	LGB testing
Compressive strength	R^2^	0.91	0.894	0.94	0.902	0.94	0.918
	MAE	2.41	2.7	2	2.4	1.8	2.4
	MSE	11.6	13.3	6.95	12.4	5.5	10.3
	FB	0	0	0	0.01	0	0
Flexural strength	R^2^	0.85	0.802	0.91	0.882	0.92	0.893
	MAE	0.54	0.66	0.4	0.51	0.35	0.52
	MSE	0.51	0.88	0.26	052	0.24	0.47
	FB	0	− 0.01	0	0.002	0	0.005
Tensile strength	R^2^	0.95	0.935	0.94	0.912	0.93	0.911
	MAE	0.28	0.3	0.25	0.29	0.23	0.32
	MSE	0.15	0.18	0.12	0.24	0.1	0.25
	FB	0	− 0.007	0	− 0.03	0	− 0.03

### Tree-based ML predictions

Accordingly, FB of all models indicates that the predictions are not severely over or underestimated. Except for the lower accuracy obtained for DT when predicting the flexural strength of BFRC, all other models reached a good prediction accuracy. DT for compressive strength reached an R^2^ of 0.91 for training and 0.894 for testing whereas GB achieved 0.94 for training and 0.902 for testing. The LGB model has outperformed both DT and GB models by achieving an R^2^ of 0.94 for training and 0.918 for testing. The MSE and MAE of the LGB model are 10.3 and 2.4 for testing, respectively.

For the flexural strength predictions, both GB and LGB showcased comparable performance by achieving an R^2^ of 0.882 and 0.893 for testing, respectively. As a result of comparatively lower performance, DT has obtained an MSE value of 0.88 for the test set which is approximate twice the value observed for LGB. A similar observation is made during the training process of MSE where the MSE of DT reached 0.51 and the remaining models reached 0.24–0.26.

From the models generated to predict tensile strength, DT has surprisingly obtained the highest accuracy. For example, the training accuracy reached an R^2^ of 0.95 for training and 0.935 for testing at a depth of 5. Both GB and LGB reached a comparable accuracy for predicting the tensile strength of BFRC. For testing, GB obtained an R^2^ of 0.912 and LGB obtained an R^2^ of 0.911. The MSE observed for DT for testing is 25% lower compared to the observed MSE values for GB and LGB.

**Figure 2:**
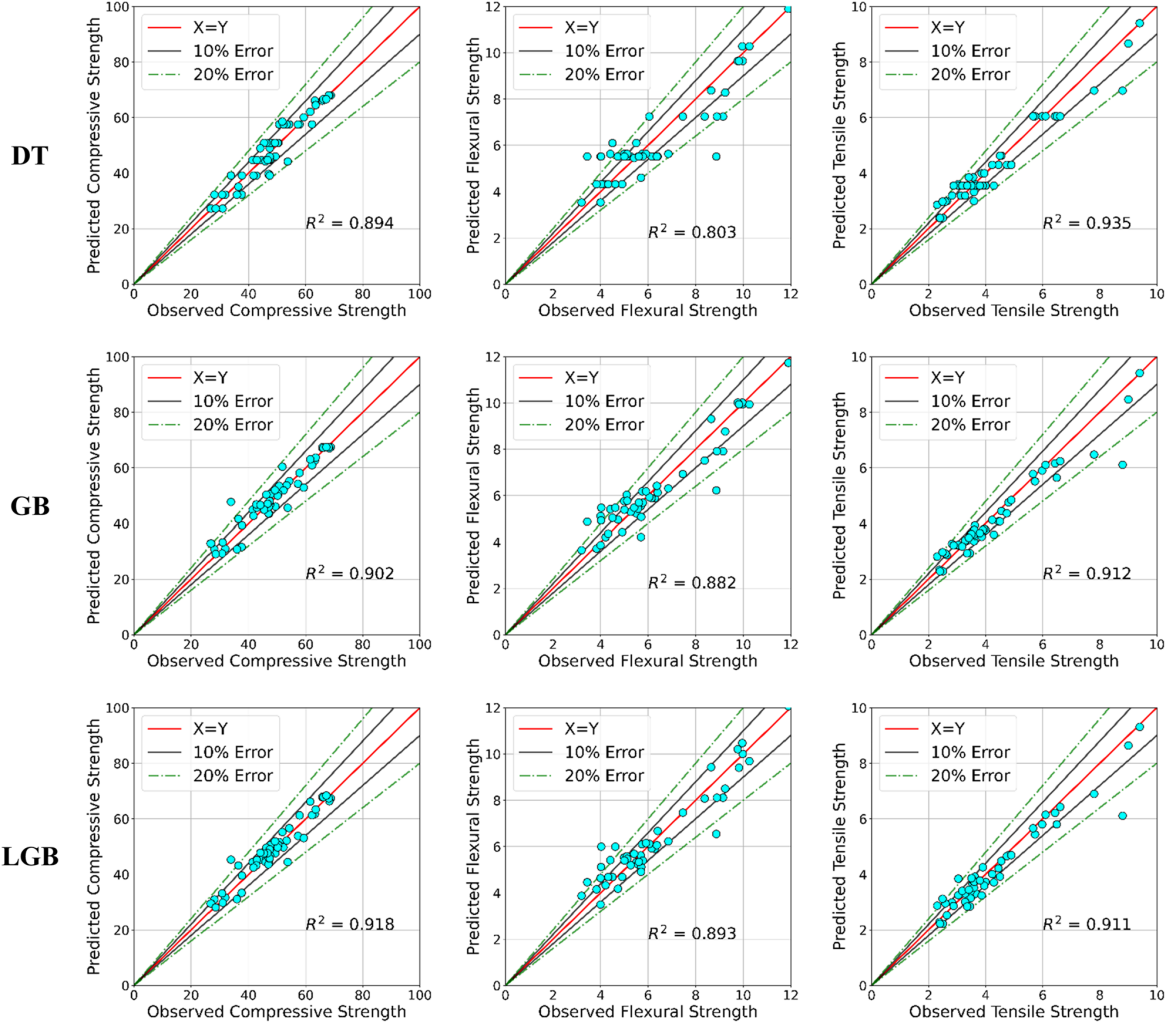
Comparison of testing predictions obtained from tree-based models.

Figure 2 shows the predictions (test) obtained from tree-based models for the strength characteristics of BFRC. For compressive strength, DT achieved an R^2^ of 0.894 whereas two gradient-boosting models (GB and LGB) achieved 0.902 and 0.918 respectively. All three models accurately predicted higher compressive strength values (> 60 Mpa). Few deviations are shown in DT predictions that lead to comparatively lower accuracy compared to gradient boosting models. However, both gradient-boosting models showcase points that deviated more than 20% compared to the original predictions.

DT obtained an R^2^ of 0.803 for flexural strength predictions. Two gradient-boosting tree models reached R^2^ of 0.882 and 0.893, respectively. As DT is the basic tree structure with step functions, the variation looks justifiable. For example, DT provides constant predictions for a given range of independent variables. Generally, this behavior diminishes when the tree depth is increased. However, on this occasion, the increase in tree depth leads to overfits. Gradient boosting tree models reached higher accuracy compared to DT. Both GB and LGB models accurately predicted higher flexural strength values (> 6 MPa) within a 10% error margin. Both models have slightly overestimated flexural strength values compared to the flexural strength values which are less than 6 MPa. Even though both GB and LGB are based on DT structure, the implementation of gradient boosting showcased a different learning (training) method.

Besides, DT obtained the highest accuracy for tensile strength predictions. The variation exhibits that the derivations are almost within a 10% error margin with respect to observed values. Gradient boosting tree models reached comparable R^2^ values of 0.912 and 0.911. both models showcased slight deviations beyond the 20% error margin which reduces their R^2^ value compared to DT. However, all three tree-based models showcased good accuracy in predicting the strength characteristics of BFRC.

By considering the performance of all three models, we decided to explain all three models using explainable AI which was a major objective of this study. Because the authors strongly believed that the AI explanations are algorithm dependent. For example, different algorithms will have different explanations as a result of unique features within the algorithm, even though they achieved comparable accuracies. In addition, the explanation is XAI-dependent. As summarized in Table 1 of this manuscript, many researchers used only a single explanation method and a single ML algorithm which overlooks the point we emphasize. In this study, we implemented both SHAP and LIME for all three tree-based models.

### XAI for tree-based models

This section provides the SHAP and LIME explanations obtained for tree-based models. The explanations can be categorized as global (model-in-whole) and local explanations (Instance-based). SHAP provides both global and local explanations whereas LIME only provides local explanations. It is recognized that SHAP provides a unified measure of feature importance, unlike LIME which works based on dummy instances.

#### Global explanations

**Figure 3:**
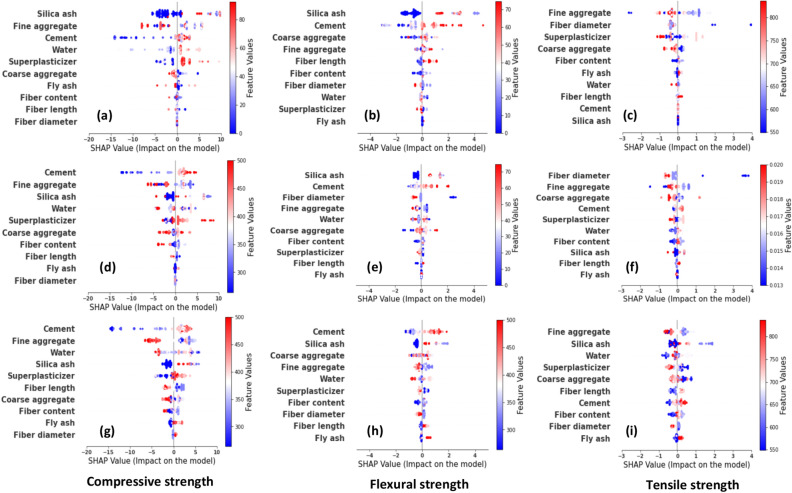
Global explanations obtained for tree-based models; (**a**–**c**) DT, (**d**–**f**) GB, (**g**–**i**) LGB.

Global explanations obtained for SHAP are exhibited in Fig. 3. For the compressive strength, DT has chosen Silica ash as the dominant variable. An increase in silica ash improves the compressive strength or vice versa. However, there are cases where higher Silica ash content has reduced the compressive strength. DT ranked fine aggregates and cement as the next dominant features, respectively. Fine aggregate content had a mixed impact on compressive strength however, higher fine aggregates lower the compressive strength. DT well captured the effect of cement on compressive strength whereas, higher cement content increases the compressive strength and lower cement content reduces compressive strength. The feature importance of water has not been well distributed. For example, Fig. 3a shows an almost neural effect from higher and lower water content which generally contradicts what is observed in compressive strength variation. For superplasticizers, higher content increases the compressive strength which agrees with the actual behavior. The remaining features; coarse aggregate, fly ash, basalt fiber content, fiber length, and diameter had less impact on the compressive strength.

From the GB algorithm, cement content had the highest feature importance ranked by SHAP. It is comparable to the explanation of the LGB model. Both GB and LGB models ranked fine aggregate as the second dominant variable. The impact observed for fine aggregate is comparable with the impact observed from the DT explanation in Fig. 3a. GB ranks silica ash and water as the next important variables whereas LGB ranks water and silica ash, respectively. Despite the order, their feature importance is comparable and agrees with general concrete behavior. For example, an increase in water content decreases compressive strength. This was different from the neural feature importance observed for Water in the DT model. All three models ranked 5th variable as superplasticizers. LGB noticed a moderately significant impact from the fiber length compared to GB and DT. Accordingly, the increase in fiber length decreases compressive strength. All three models agree that fiber diameter is the least significant feature.

Silica Ash is the dominant variable for flexural strength according to DT. An increase in silica ash improves the flexural strength and lowering silica ash decreases the flexural strength. However, in some instances, a lower flexural strength has resulted from a higher silica ash content. Subsequently, cement and coarse aggregate are the dominant variables ranked by DT. It is highlighted that a higher cement content decreased flexural strength. Fine aggregates demonstrate an almost neutral flexural strength variation. In terms of basalt fiber characteristics, less feature importance is observed on the flexural strength.

Repeatedly, silica ash content received the highest feature importance for flexural strength from the GB algorithm. However, the GB model ranked cement second whereas the LGB model ranked cement first. Silica ash was ranked second in the LGB model. The impact observed for fine aggregate in GB and LGB is comparable to the DT explanation (Fig. 3b,e,h). Thereupon, LGB ranks water and silica ash as the subsequent important variables, whereas GB ranks silica ash and water. The SHAP explanation depicts that the flexural strength decreases as fiber diameter increases. Fly ash was the least significant feature ranked by tree-based models.

From the explanations on tensile strength, DT and LGB have ranked fine aggregates as the dominant variable. It is important to notice that DT and GB identify a higher feature importance from fiber diameter which disagrees with the LGB model. Lowering fiber diameter has increased the tensile strength of BFRC. It is observed that higher fine aggregate content lowers the tensile strength. Unlike in compressive and flexural strength, cement and water content have fairly lower importance on tensile strength. Interestingly, all three models exhibit that the higher superplasticizer content has lowered the tensile strength though it increased the compressive strength. Except for a few cases, basalt fiber properties had relatively less impact on strength characteristics compared to the remaining variables. DT selected silica ash as the least important factor for tensile strength where as it was chosen as the highest importance in flexural strength and compressive strength. GB and LGB have ranked fly ash as the least important variable.

Therefore, the authors highlight that previous studies on machine learning interpretability did not capture this uniqueness in algorithms. For example, they used mostly SHAP on their best-performed model whereas we improved all the models to the same good performance and used SHAP and LIME. These algorithms have a critical role in the explanation. How an algorithm learns the patterns will depend on their inner implementations. For example, the GB and LGB model most of the time showcased comparable explanations as it is based on gradient boosting. On the other hand, such differences in explanation make the decision criteria complicated.

**Figure 4:**
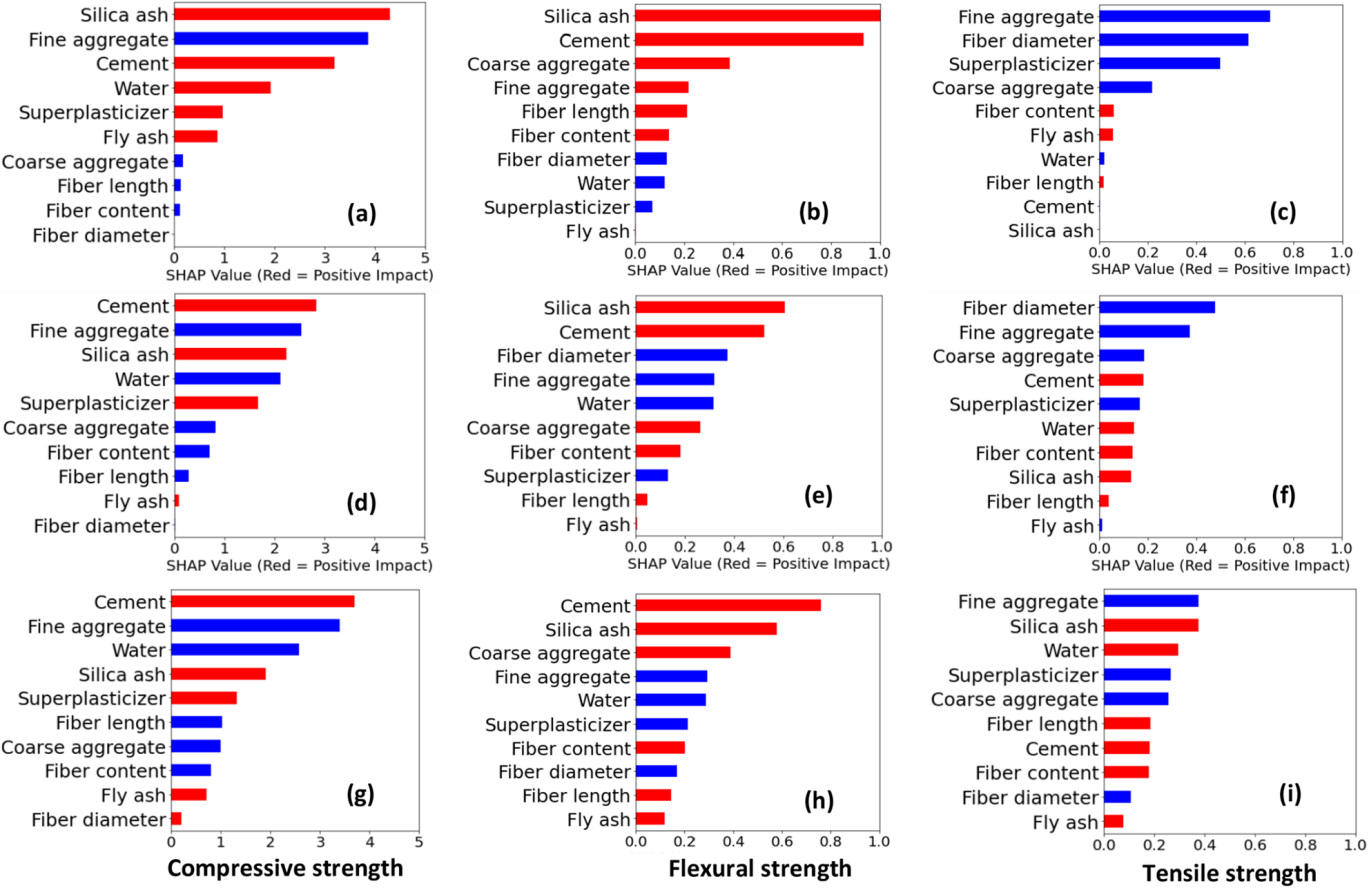
Mean absolute SHAP (global) explanations obtained for tree-based models; (**a**–**c**) DT, (**d**–**f**) GB, (**g**–**i**) LGB.

Figure 4 illustrates the average feature importance for each regression model. Red color indicates positive impacts and blue color represents negative impacts. For compressive strength, the average feature importance values obtained from DT are considerably different with respect to GB and LGB. For example, DT identified both negative and positive impacts from the water content which contradicts actual observations. However, GB and LGB showcase a negative impact from water content. Also, both GB and LGB have ranked features in the same manner (positive/negative) despite their magnitude. LGB has given slightly higher feature importance values. Furthermore, fly ash has a non-negligible, positive effect on the compressive strength, observed in DT and LGB explanations which do not agree with the GB model. DT has given the least feature importance for fiber length, diameter, and content. Both GB and LGB have given a certain (negative) importance level for fiber content and fiber length.

A significant difference is observed in the explanations observed for flexural strength. For example, the DT explanation provides that all top six ranked variables have a positive impact on flexural strength. That disagrees up to a certain extent with the explanation observed in GB and LGB for fine aggregates. Not only the order of feature importance but also their magnitudes are also different for each regression. Similar observations are made for the tensile strength. As highlighted in the global explanation previously, there exists a negative impact from superplasticizers on the flexural and tensile strength. All three models emphasized overall a negative impact from fiber diameter, coarse and fine aggregates, and superplasticizers on the tensile strength. GB and LGB showcased a positive impact from water content on the tensile strength whereas DT showcased a negligible (negative) impact.

#### Feature dependencies

**Figure 5:**
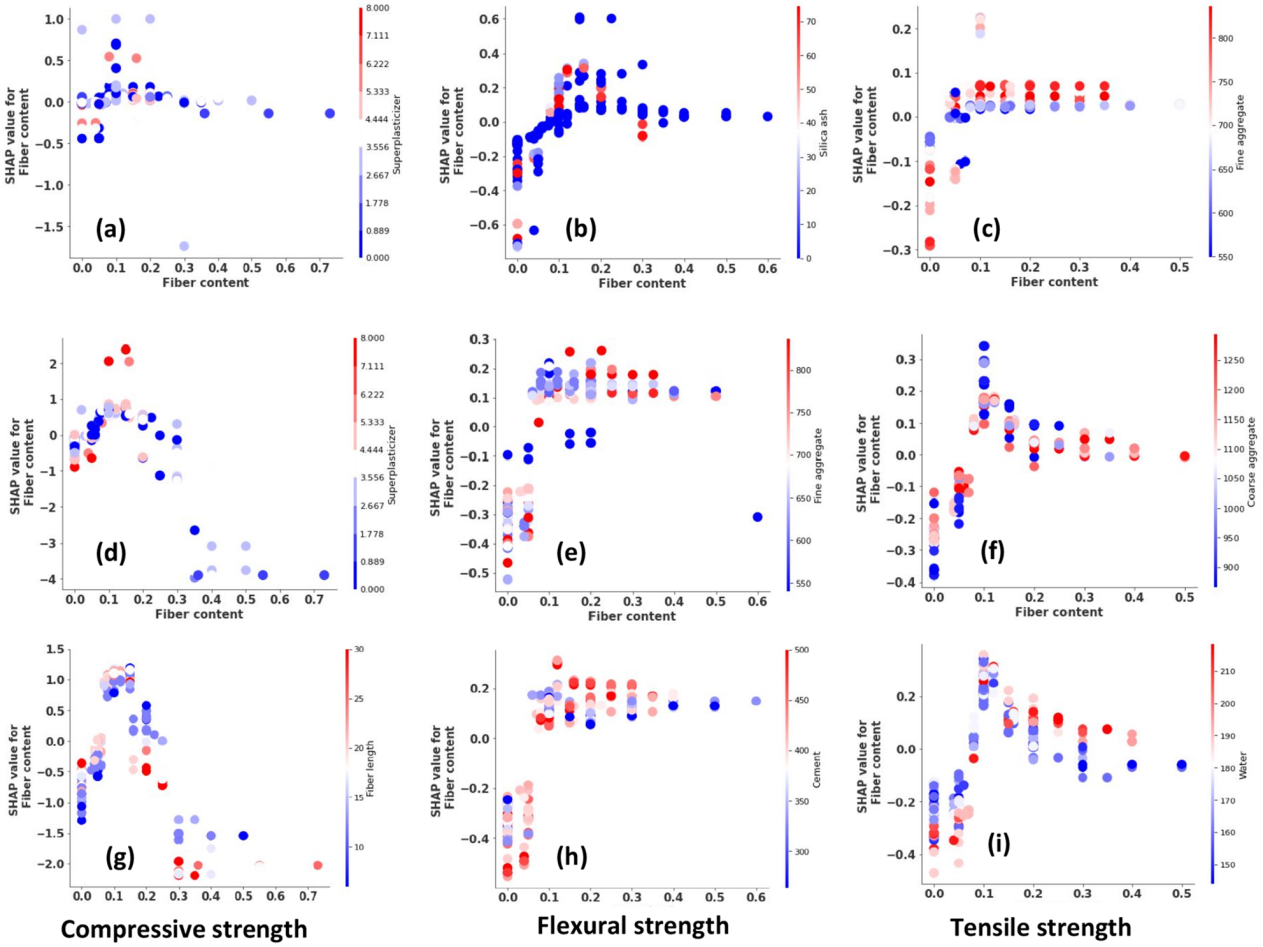
Dependency plots for basalt fiber content; (**a**–**c**) DT, (**d**–**f**) GB, (**g**–**i**) LGB.

Apart from global explanation, SHAP provides the interaction of each feature as well. Figure 5 shows the feature dependency plots obtained for basalt fiber content. Except for its lower feature importance compared to other constituents, it has an optimum content at which the maximum improvement in each strength characteristic is observed. This dependency provides a greater picture of how the feature contributes to the output and with which the feature has mostly interacted. All three strength characteristics showcase an optimum at 0.1–0.2 fiber content. The colour scale defines the variable that the fiber content mostly interacts with. For example, both DT and GB decide the fiber content is mostly associated with superplasticizer content whereas LGB decides it is the fiber length instead of superplasticizer content. Blue and red colours indicate how the fiber content interacts with the range of given values of the second parameter. According to GB, the SHAP value for fiber content is high when it interacts with higher (Red colour dots) superplasticizer content.

For flexural strength, GB and LGB exhibit comparable dependencies despite the interacted variable. GB decided the interacted variable as fine aggregates and LGB select cement content. However, higher fine aggregate or cement content is mostly associated with fiber contents ranging from 0.1 to 0.4 always increases flexural strength. On the other hand, lower silica content associated with basalt fiber content ranges from 0.1 to 0.3 mostly increasing the flexural strength. Based on DT, the SHAP has given a large impact on fiber content compared to GB and LGB models. According to the DT model, an increase in fiber content improves the tensile strength and the variation become stalled after the fiber content reached 0.2. Mostly, the impact of fiber content is high when it interacts with higher fine aggregates as shown in Fig. 5. GB and LGB displayed that the fiber content is mostly associated with coarse aggregates and water content, respectively. SHAP value for fiber content becomes high for lower coarse aggregates as shown in the plot observed for GB. LGB depicts a mixed variation in the water content.

#### Local explanations

**Figure 6:**
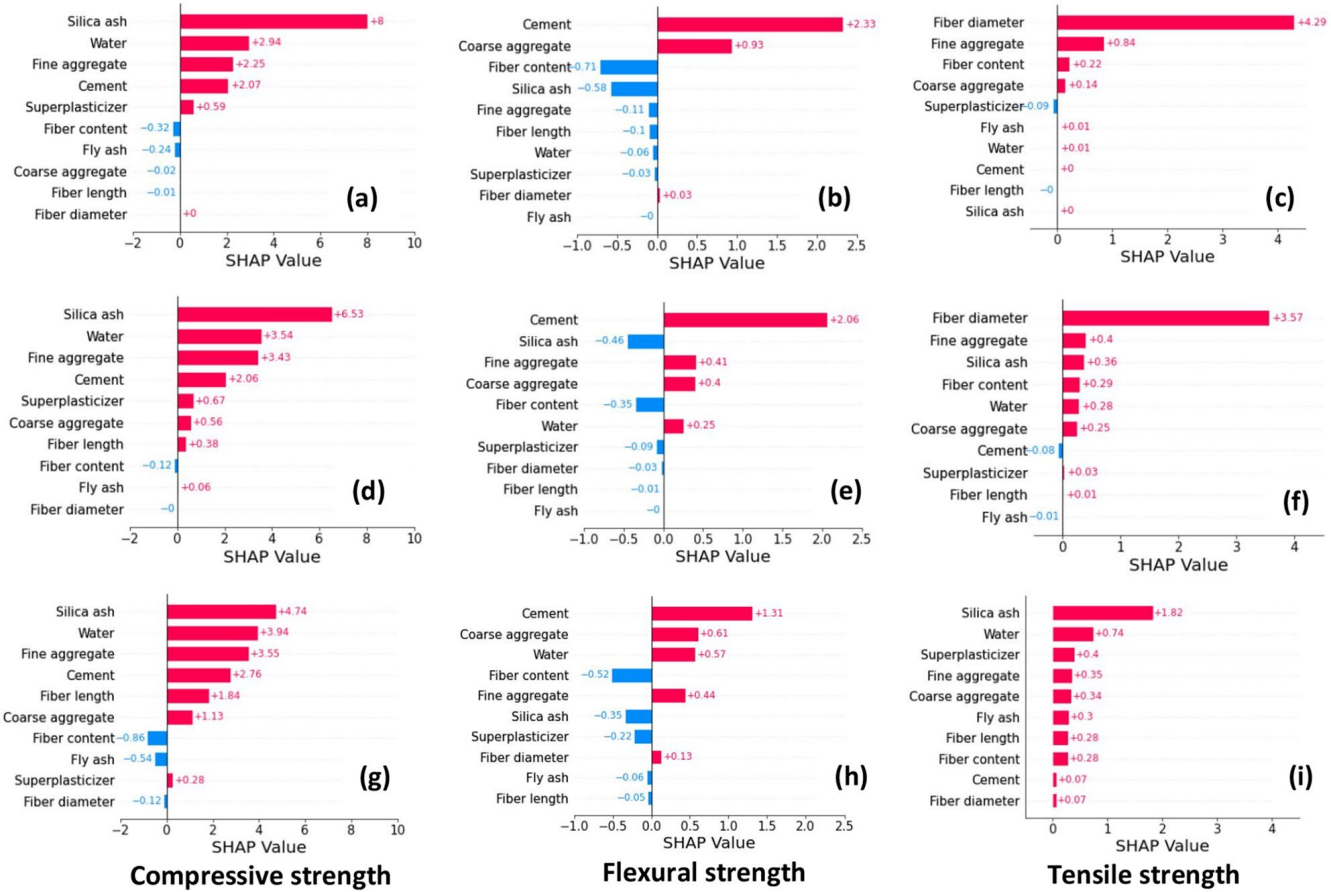
SHAP local explanations; (**a**–**c**) DT, (**d**–**f**) GB, (**g**–**i**) LGB.

Figure 6 represents the SHAP local explanations observed for higher strength values in each strength characteristic. The authors picked a random value from each strength and explained it using both SHAP and LIME. SHAP's local importance does not always need to agree with global importance. For example, in all three cases for compressive strength, silica ash had the highest feature importance. In the SHAP scale, the feature importance for silica ash is 8 for DT, 6.53 for GB, and 4.74 for LGB. Moreover, DT and GB have given a considerably higher feature importance for silica ash compared to the remaining parameters. Interestingly, all three tree-based models ranked the first four features of the instance in the same order. For instance, fiber content and fly ash content have negatively contributed.

For the instance selected from flexural strength, tree-based models identified cement as the dominant variable. For the second dominant feature, both DT and LGB identified coarse aggregates whereas the negative impact of silica ash has been highlighted. Subsequently, DT indicates a negative contribution from fiber content, silica ash, fine aggregates, fiber length, water, and superplasticizer. However, GB and LGB show a positive contribution from the fine aggregates and water content that contradicts with DT explanation.

Fiber diameter contributes most to the instance obtained from tensile strength, according to GB and DT. SHAP has given a feature importance of 4.29 and 3.57 for the fiber diameter in DT and GB, respectively. Nevertheless, the LGB model contradicts this by ranking the fiber diameter as the least important feature. Overall, all features positively contribute to the selected instance of tensile strength.

**Figure 7:**
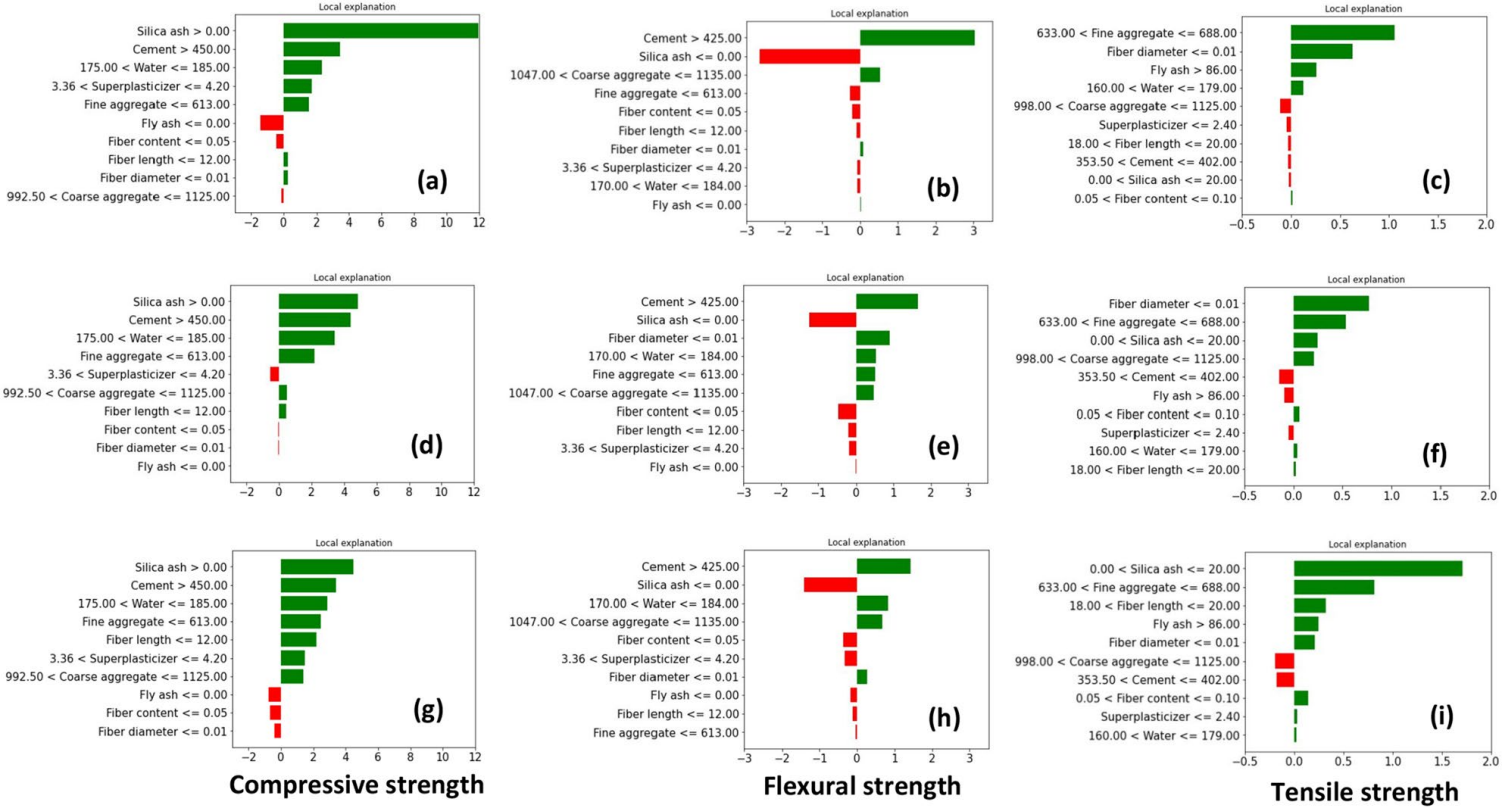
LIME local explanations; (**a**–**c**) DT, (**d**–**f**) GB, (**g**–**i**) LGB.

LIME weighs an instance by creating dummy instances, therefore, would not reflect the actual instance. Figure 7 shows the feature importance obtained from the LIME explanation for the same instances explained in Fig. 6. Accordingly, silica ash dominates the instance picked from compressive strength. LIME provides a logical explanation based on the feature value. For instance, silica ash > 0, cement content > 450, 175 < water content < 185, and fine aggregates < 613 had a positive contribution to the compressive strength.

LIME has provided a comparable feature importance for silica ash and cement for the flexural strength value predicted from each regression model. However, the feature importance gradually drops from the DT to LGB. LIME decides that silica ash content lower than 0 would contribute negatively to flexural strength. All three models emphasize that fiber diameter ≤ 0.01 had a slightly positive impact and fiber length ≤12 had a negative impact on the selected instance.

LIME explanation obtained for tensile strength highlighted different feature importance order. The highest important feature was fine aggregates for DT, fiber diameter for GB, and silica ash content for LGB. All of those dominant features had a positive impact on the selected instance. Both DT and LGB agree with a positive contribution from fly ash > 86 and contract with the same but negative contribution displayed by the GB model. Gradient boosting models have obtained a negligible feature importance for water content (160 < water content < 180) whereas a moderate feature importance was given in the DT model.

### User-friendly computer application

**Figure 8:**
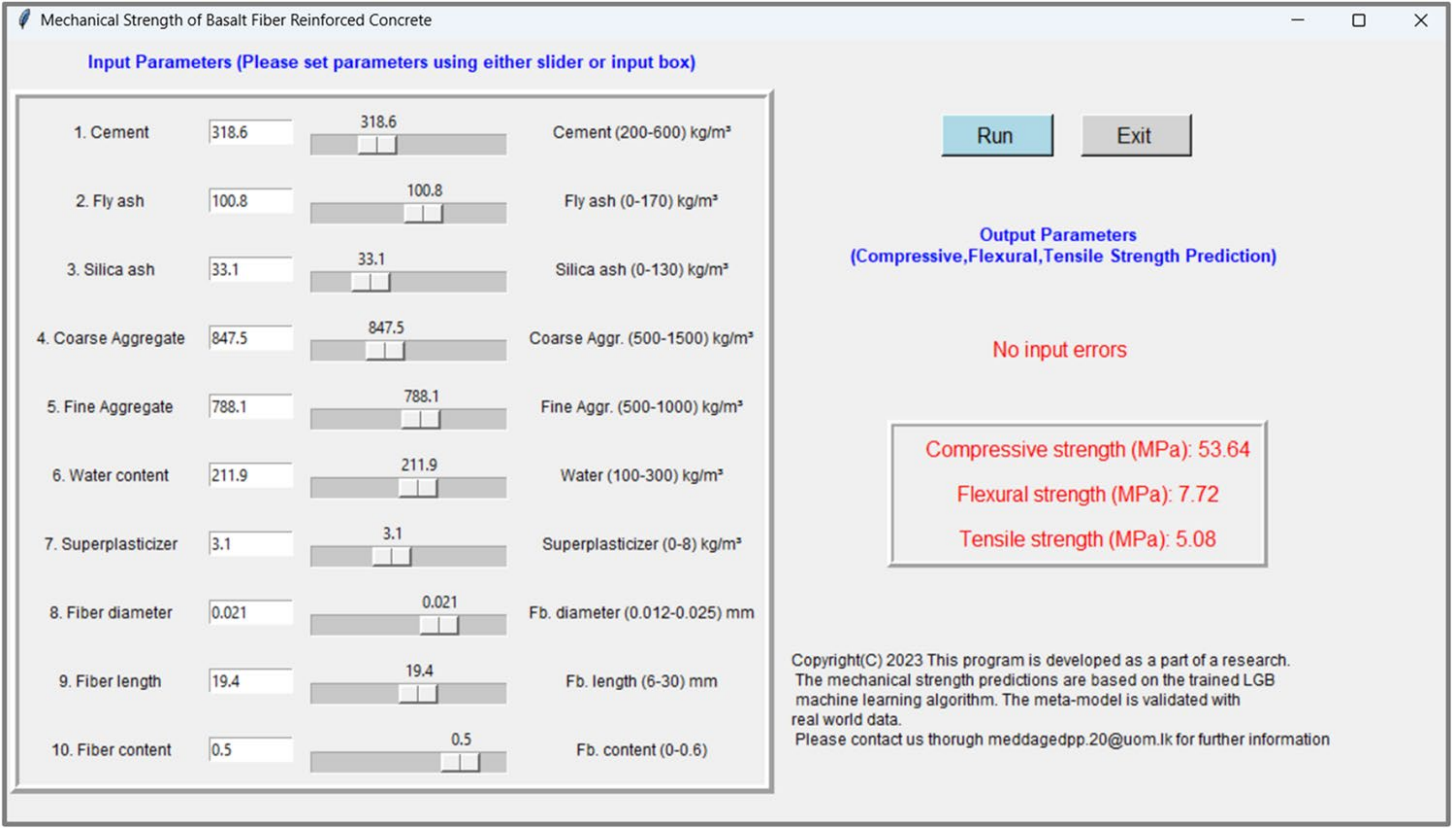
GUI-based application developed to predict strength characteristics of BFRC.

A user-friendly computer application to predict compressive, flexural, and tensile strength was developed based on the LGB model. Since the training and testing splits verified the applicability of the LGB model (R^2^ > 0.89 in all cases compared to the remaining models), the whole data set was simultaneously used for the training final models. As the whole data set is employed, the depth of the LGB model was increased to six by keeping the remaining hyperparameters constant. Three LGB models were written into GUI and they achieved an R^2^ > 0.95 learning phase (with the whole data set). The graphical user interface (GUI) is shown in Fig. 8 of the developed application. This application enables users to input ten parameters (Cement content, fly ash content, water content, etc.) including three parameters of Basal fibers (diameter, length, content). The error handling capability of the proposed GUI ensures the user is directed to input values within acceptable range and obtain mechanical strength characteristics. The authors believe that this application will provide a convenient and efficient method of predicting strength parameters while enabling different parametric studies on this concrete technology. For more precise prediction the application guide users to limit input parameters to the range in which the LGB model was fitted.

### Limitations of the study

This study specifically assessed the explanations obtained from different explainable AI models on different ML models. With a limited dataset, explanations tend to be algorithm dependent rather than data dependent. Therefore, the authors strongly recommend the use of domain experts-based surveys or trials on explanations to investigate their fidelity. Even though SHAP and LIME are widely used black-box explanations, they do occasionally consist of moderate contradictions in explanations.

## Conclusion

This study used tree-based machine learning algorithms to model the strength characteristics (compressive, flexural, and tensile) of basalt fiber-reinforced concrete. Subsequently, we used interpretable machine learning (SHAP, and LIME) to elucidate all models locally and globally. Finally, we developed a ready-to-use tool with a convenient Graphical User Interface (GUI) to predict the strength characteristics of basalt fiber-reinforced concrete. The conclusion of the study can be summarized as follows,

- Tree-based regression models can capture the non-linear behavior of strength characteristics of Basalt fiber reinforced concrete DT models reached a training R^2^ > 0.85 and testing R^2^ > 0.802, GB models reached a training R^2^ > 0.91 and testing R^2^ > 0.882 for predicting strength characteristics, and LGB models reached a training R^2^ > 0.92 and testing R^2^ > 0.89 in all cases.
- Although all the models demonstrated good predictive accuracy, the LGB model performed slightly better than the other two models. As a result, the LGB model is a suitable option for precise forecasting of the strength properties of basalt fiber reinforced concrete. However, it does not rule out the applicability of DT and GB tree models to predict the mechanical strength of BFRC.
- For the first time in concrete research studies, model interpretability was obtained using SHAP and LIME explanation methods. SHAP provided detailed resolution on model-in-whole, instances, and dependencies of features whereas LIME provided instance-based explanations. They converted the black-box nature of the ML models to a glass box (interpretable) by revealing the causality of predictions.
- For the first time, we used explainable AI on all three models to investigate how different these models work. Interestingly, the authors observed that the explanations are algorithm dependent. Even the SHAP explanations obtained for compressive strength were unique for each regression model either by magnitude or the order of feature importance. This aspect was overlooked in previous studies as they used explainable AI on the best-performed model. The authors argue that, under the same level of accuracy, different ML algorithms can provide different explanations.
- This study further provided a graphical user interface to predict the mechanical strength of basalt fiber-reinforced concrete based on the LGB model. The GUI can efficiently predict all compressive, flexural, and tensile strengths of BFRC and will be helpful to the research community which investigates the use of ML related to BFRC.

## Supplementary Information

Supplementary Information.

## Data Availability

Data will be made available on request from the corresponding author.

## Abbreviations

ADABoost: Adaptive boosting
ANFIS: Adaptive neuro-fuzzy interference system
ANN: Artificial neural network
BFRC: Basal fiber reinforced concrete
CART: Classification and regression tree
CNN: Convolution neural network
CSA: Coupled simulated annealing
DT: Decision tree
EBM: Explainable boosting machine
ELM: Extreme learning machine
ET: Extra tree
FB: Fractional bias
FRP: Fiber-reinforced plastic
GB: Gradient boosting tree
GEP: Gene expression programming
GPR: Gaussian process regression
GUI: Graphical user interface
KELM-GA: Kernel extreme learning machine with genetic algorithms
LASSO: Least absolute shrinkage and selection operator
LGB: Light gradient boosting
LIME: Local interpretable model agnostic explanation
LKRR: Laplacian kernel ridge regression
LSSVM: Least square support vector machine
MAE: Mean absolute error
MSE: Mean squared error
MLR: Multiple linear regression
R: Coefficient of correlation
R^2^: Coefficient of determination
RF: Random forest
SHAP: Shapley additive explanation
SSE: Sum of square error
SVR: Support vector regression
UPV: Ultrasonic pulse velocity
XAI: Explainable artificial intelligence
XGB: Extreme gradient boosting
