# Supplementary material for: Modeling strength characteristics of basalt fiber reinforced concrete using multiple explainable machine learning with a graphical user interface
Source: Sci Rep. 2023 Aug 12;13:13138. doi: 10.1038/s41598-023-40513-x (PMC10423212; doi:10.1038/s41598-023-40513-x)
Supplement: Supplementary file 1 — Supplementary Information. [file 41598_2023_40513_MOESM1_ESM.docx]

**Annexure**

**Annex A1:** Descriptive statistics of three datasets (St.d represents standard deviation)

|  | **Cement**  **(Kg/m^3^)** | **Fly ash (Kg/m^3^)** | **Silica ash**  **(Kg/m^3^)** | **Coarse aggregate**  **(Kg/m^3^)** | **Fine aggregate**  **(Kg/m^3^)** | **Water**  **(Kg/m^3^)** | **Superplasticizer**  **(Kg/m^3^)** | **Fiber diameter**  **(mm)** | **Fiber length**  **(mm)** | **Fiber content** | **Strength (Mpa)** |
| --- | --- | --- | --- | --- | --- | --- | --- | --- | --- | --- | --- |
| Compressive strength data | | | | | | | | | | | |
| **count** | 267 | 267 | 267 | 267 | 267 | 267 | 267 | 267 | 267 | 267 | 267 |
| **mean** | 401 | 45 | 13 | 1079 | 704 | 176 | 2.9 | 0.0 | 16.6 | 0.124 | 49.5 |
| **St.d** | 76 | 57 | 29 | 177 | 109 | 31 | 1.9 | 0.0 | 5.9 | 0.118 | 11.4 |
| **min** | 217 | 0 | 0 | 512 | 507 | 112 | 0.0 | 0.0 | 6.0 | 0.000 | 23.3 |
| **25%** | 350 | 0 | 0 | 993 | 613 | 160 | 1.1 | 0.0 | 12.0 | 0.050 | 41.8 |
| **50%** | 402 | 0 | 0 | 1125 | 690 | 175 | 3.4 | 0.0 | 18.0 | 0.100 | 48.6 |
| **75%** | 450 | 86 | 0 | 1181 | 787 | 185 | 4.2 | 0.0 | 20.0 | 0.160 | 59.1 |
| **max** | 613 | 168 | 126 | 1540 | 1194 | 301 | 8.0 | 0.0 | 30.0 | 0.730 | 69.9 |
| Flexural strength data | | | | | | | | | | | |
| **count** | 245 | 245 | 245 | 245 | 245 | 245 | 245 | 245 | 245 | 245 | 245 |
| **mean** | 394 | 29 | 13 | 1118 | 691 | 175 | 3.1 | 0.0 | 16.6 | 0.139 | 6.5 |
| **St.d** | 74 | 44 | 25 | 174 | 110 | 33 | 2.4 | 0.0 | 6.3 | 0.119 | 2.3 |
| **min** | 217 | 0 | 0 | 512 | 507 | 112 | 0.0 | 0.0 | 6.0 | 0.000 | 2.4 |
| **25%** | 346 | 0 | 0 | 1047 | 613 | 160 | 0.0 | 0.0 | 12.0 | 0.050 | 4.9 |
| **50%** | 400 | 0 | 0 | 1135 | 671 | 170 | 3.4 | 0.0 | 17.0 | 0.100 | 5.7 |
| **75%** | 425 | 60 | 20 | 1189 | 758 | 184 | 4.2 | 0.0 | 20.0 | 0.200 | 8.1 |
| **max** | 613 | 168 | 126 | 1540 | 1194 | 301 | 8.4 | 0.0 | 30.0 | 0.600 | 13.6 |
| Tensile strength data | | | | | | | | | | | |
| **count** | 267 | 267 | 267 | 267 | 267 | 267 | 267 | 267 | 267 | 267 | 267 |
| **mean** | 403 | 46 | 16 | 1080 | 698 | 178 | 3.3 | 0.0 | 16.7 | 0.127 | 134.0 |
| **St.d** | 74 | 56 | 31 | 162 | 88 | 30 | 2.2 | 0.0 | 6.4 | 0.106 | 77.2 |
| **min** | 217 | 0 | 0 | 512 | 507 | 125 | 0.0 | 0.0 | 6.0 | 0.000 | 1.0 |
| **25%** | 354 | 0 | 0 | 998 | 633 | 160 | 2.4 | 0.0 | 12.0 | 0.050 | 67.5 |
| **50%** | 402 | 0 | 0 | 1125 | 688 | 179 | 4.0 | 0.0 | 18.0 | 0.100 | 134.0 |
| **75%** | 450 | 86 | 20 | 1180 | 781 | 188 | 4.8 | 0.0 | 20.0 | 0.200 | 200.5 |
| **max** | 613 | 168 | 126 | 1540 | 875 | 301 | 8.4 | 0.0 | 30.0 | 0.500 | 267.0 |

**Annex A2:** Pairwise correlation between independent and dependent features

|  | **Compressive strength** | **Flexural Strength** | **Tensile Strength** |
| --- | --- | --- | --- |
| **Cement** | 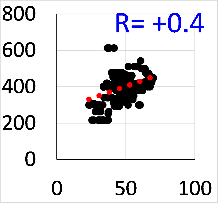 | 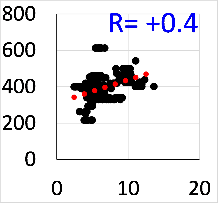 | 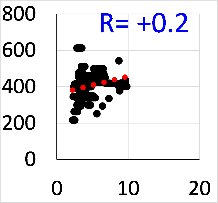 |
| **Fly Ash** | 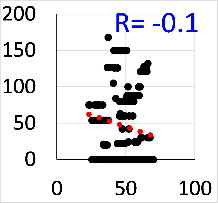 | 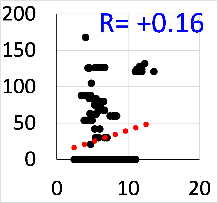 | 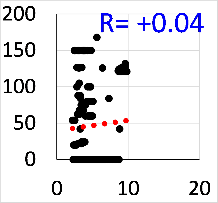 |
| **Silica Ash** | 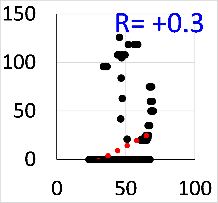 | 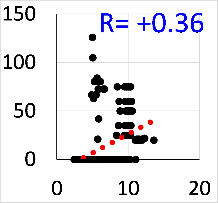 | 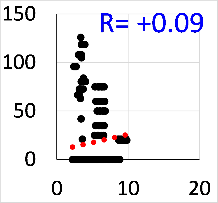 |
| **Coarse Aggregate** | 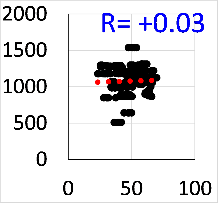 | 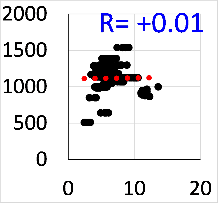 | 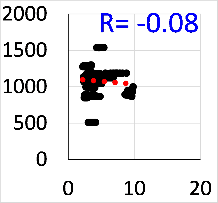 |
| **Fine Aggregate** | 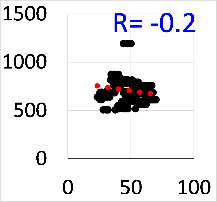 | 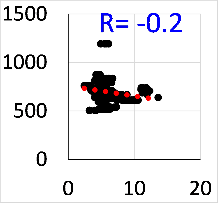 | 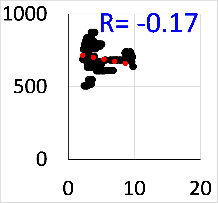 |
| **Water** | 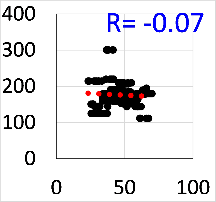 | 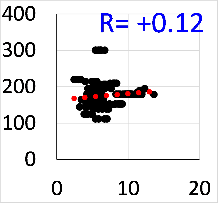 | 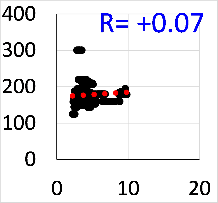 |
| **Superplasticizer** | 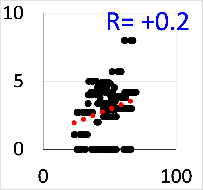 | 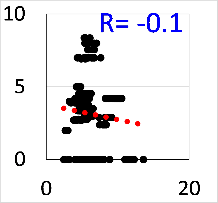 | 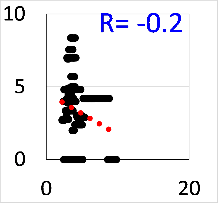 |
| **Fiber diameter** | 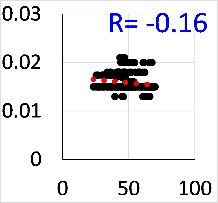 | 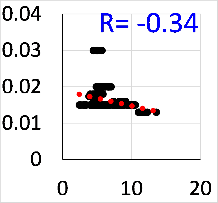 | 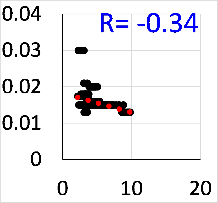 |
| **Fiber length** | 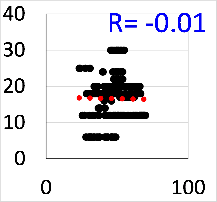 | 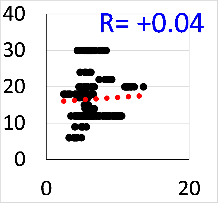 | 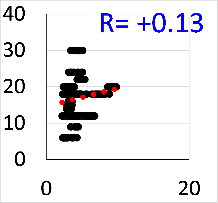 |
| **Fiber content** | 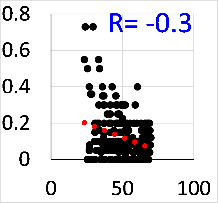 | 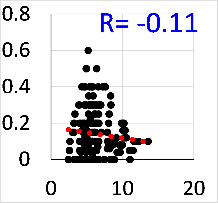 | 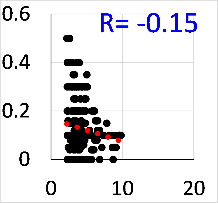 |

**Annex A3**: Optimized hyperparameters of tree-based ML models

|  | Compressive strength | | | Flexural strength | | | Tensile strength | | |
| --- | --- | --- | --- | --- | --- | --- | --- | --- | --- |
|  | DT | GB | LGB | DT | GB | LGB | DT | GB | LGB |
| Max Depth | 5 | 3 | 4 | 5 | 3 | 4 | 4 | 4 | 3 |
| Min sample leaf | 1 | 1 | - | 1 | 1 | - | 1 | 1 | - |
| Min sample split | 2 | 2 | - | 2 | 2 | - | 2 | 2 | - |
| Estimators | - | 100 | 100 | - | 100 | 100 | - | 100 | 100 |
| ccp_alpha | 0 | 0 | - | 0 | 0 | - | 0 | 0 | - |
| Splitter | best | - | - | best | - | - | best | - | - |
| Learning rate | - | 0.1 | 0.1 | - | 0.1 | 0.1 | - | 0.1 | 0.1 |
| Alpha | - | 0.9 | - | - | 0.9 | - | - | 0.9 | - |
| Reg_alpha | - | - | 0 | - | - | 0 | - | - | 0 |
| Reg_lambda | - | - | 0 | - | - | 0 | - | - | 0 |
